# Smoking and diabetes cause telomere shortening among alcohol use disorder patients

**DOI:** 10.1038/s41598-024-55195-2

**Published:** 2024-02-26

**Authors:** Shinsaku Inomata, Hiroaki Arima, Takahiro Fukuda, Hiroki Ozawa, Taro Yamamoto

**Affiliations:** 1 Graduate School of Biomedical Sciences, Nagasaki University, 1-12-4 Sakamoto, Nagasaki, Nagasaki Japan; 2 Department of International Health and Medical Anthropology, Institute of Tropical Medicine, Nagasaki University, 1-12-4 Sakamoto, Nagasaki, Nagasaki 852-8523 Japan; 3 Akiyama Hospital, 737-1, Meshiro, Isahaya, Nagasaki Japan; 4 Department of Global Mental Health Science, Nagasaki University, 1-12-4 Sakamoto, Nagasaki, Nagasaki Japan

**Keywords:** Alcohol use disorder, Telomere length, Smoking, Diabetes, Biomarkers, Medical research, Risk factors

## Abstract

The length of telomeres located at the ends of chromosomes has attracted attention as an indicator of cellular and individual aging. Various diseases or stresses cause telomere shortening, and it has been reported that alcohol use disorder patients actually have shorter telomeres than healthy patients. However, the factors that contribute to the reduction in telomere length among alcohol use disorder patients have not been clarified in detail. Therefore, in this study, we explored the factors that reduce telomere length in alcohol use disorder patients. A questionnaire survey and a measurement of leukocyte telomere length were conducted among alcohol use disorder patients. The mean telomere length of leukocyte was measured by ∆∆Ct analysis using a real-time PCR. We compared the telomere length between alcohol use disorder patients and the control group (Japanese special health check-up examinee). Moreover, we searched for factors associated with telomere length from drinking/smoking characteristics and history of comorbidities. A total of 74 subjects had alcohol use disorder, and 68 were in the control group. Compared to the control group, alcohol use disorder patients had significantly shorter telomere lengths (p < 0.001). A multivariate analysis revealed that a longer duration of smoking resulted in a significantly shorter telomere length (p = 0.0129). In addition, a comparison of the telomere length between the groups with and without a history of suffering from each disease revealed that telomere length was significantly shorter in the group with diabetes than in the group without diabetes (p = 0.0371). This study reveals that in individuals with alcohol dependence, particularly, prolonged smoking habits and the presence of diabetes contribute to telomere shortening. Medication and support for abstinence from alcohol has been mainly provided for alcohol use disorder patients. Our findings demonstrate a potential support approach via smoking cessation programs and controlling diabetes, which may be helpful to suppress the shortening of healthy life expectancy among alcohol use disorder patients.

## Introduction

Alcohol use disorder (AUD) is a mental disorder in which a person loses control over their drinking behavior, deviates from society, and repeatedly consumes alcohol^1^. In addition, AUD causes many complications, such as coronary artery disease, various cancers, and dementia^2^. According to the World Health Organization (WHO), alcohol consumption accounted for 5.3% of all deaths worldwide in 2016, surpassing the rates associated with hypertension and diabetes^3^. Based on a 2020 report, approximately 80% of the world’s population has consumed alcohol in their lifetime, with Iraq having the lowest rate of alcohol consumption at 3.8% and Peru having the highest rate at 97.1%^4^. The accurate data regarding post-COVID prevalence rates of alcohol consumption and alcohol dependence have not been globally aggregated. However, as of 2022, Alcohol-Related Liver Disease (ARLD) has been reported at 4.8% worldwide^5^. While there are variations in the aggregation methods, the prevalence of AUD in Japan estimated from data over the past month is reported to be 3.4%. Nonetheless, it is estimated that only about 70% of heavy drinkers perceive their drinking habits as problematic^6^. In recent years, it has also been noted that AUD patients may have shorter telomeres than healthy individuals^7–9^. Furthermore, it has been reported that human cells undergo changes in the expression of telomere-related proteins and enzymes, as well as alterations in DNA methylation patterns, due to ethanol^10^. Telomeres are DNA regions located at the ends of chromosomes, and it is known that their length decreases due to aging and various diseases; therefore, telomere length is considered a biomarker representing healthy life expectancy^11^. For example, it has been reported that telomeres are shorter in arteries and mononuclear cells of diabetic patients^12^. On the other hand, it has not been clarified what factors actually shorten telomere length in AUD patients.

## Objective

In this study, one of the objectives is to analyze telomere length among various patients, not only those hospitalized with AUD but also those participating in self-help group after undergoing treatment programs. By including patients from post-treatment self-help group, the aim is to explore the effects of abstinence periods and lifestyle habits on telomere length across a diverse patient population. The study attempts to investigate not only the direct impact of alcohol consumption on telomeres but also the effects of common lifestyle habits and comorbidities prevalent among individuals with AUD. This approach aims to explore factors contributing to reduced lifespan among individuals with AUD from a broader perspective.

## Methods

### Study population

From March 2021 to November 2021, we conducted a questionnaire survey and sample collection. We explained this study to AUD patients who had been treated at Mimatsu Medical Corporation Akiyama Hospital, Seicho Medical Corporation Sanwa Chuo Hospital, Kosei Medical Corporation Michino Hospital and National Hospital Organization Hizen Psychiatric Center. Similarly, we recruited people who had participated in the specified nonprofit corporation Yamanashi self-help group and general incorporated association Fukuoka self-help group. A self-help group is an organization where AUD patients gather to talk about their experiences and work together to remain abstinent from alcohol. Hospitalized patients and participants in the self-help group are collectively referred to as the alcohol use disorder group (AUDG). We also recruited specific health checkup participants in Matsuura City from June 18 to July 2, 2021, as the control group (CONT). To adjust each generation population (20 s, 30 s, 40 s, 50 s, 60 s, and 70 s and older) of CONT for that of AUDG, the target ID of subjects in CONT were randomly selected by R studio (runif function). This study was conducted in accordance with the provisions of the Declaration of Helsinki.

### Questionnaire survey

A questionnaire survey was administered targeting patients. We asked about basic attributes such as age, sex, height, and weight, and we calculated body mass index (BMI). For drinking characteristics, we asked about the ages at which drinking started, heavy drinking started, heavy drinking ended, and the duration of abstinence. In addition, regarding smoking habits, we asked the age at first smoking experience, the number of cigarettes smoked per day, and the duration of smoking. We calculated the heavy drinking duration, abstinence duration, Brinkman Index, and smoking duration. Furthermore, we assessed the history of diabetes, hypertension, gastroduodenal disease, biliary-hepato-pancreatic disease, and cancer. The Brinkman index involved multiplying the number of cigarettes smoked per day by the duration of smoking in years to calculate^13^.

### Measurement of telomere length

Two milliliters of whole blood was collected from the subjects into ethylenediaminetetraacetic acid-supplemented blood collection tubes and stored at − 20 °C until DNA extraction. Ten days after blood collection, DNA was extracted using the QIAamp DNA Mini Kit (Qiagen, Hulsterweg, Netherlands). Real-time PCR was performed according to previously reported primers, reagents, and reaction conditions^14^. QuantStudio 3 (Thermo Fisher Scientific, MA, USA) was used as a real-time PCR device, and the template DNA was adjusted to 25 ng or more. Additionally, the T/S ratio was calculated by the obtained Ct value, and the telomere length of the sample was calculated by the relative ratio to a reference human gDNA sample (ScienCell Research Laboratories, CA, USA) for which the absolute telomere length was known.

### Statistical analysis

We compared telomere length between AUDG and CONT by the Wilcoxon rank sum test. We plotted the relationship between telomere length and each factor and analyzed the maximum information coefficient (MIC). Furthermore, in consideration of the effect of age on telomere length, a generalized linear model analysis was performed using age and each factor as explanatory variables and telomere length as the objective variable. In addition, the Wilcoxon rank sum test was used to compare the telomere length between the group with and without a history of each visceral disease. The Wilcoxon rank sum test was used to compare ages in groups with and without a history of each disease to determine if there was an association between disease history and age. Since AUD and smoking have been reported to increase the risk of developing diabetes^15^, we compared the duration of heavy drinking, the Brinkman Index, and the smoking duration between those with and without a history of diabetes. Furthermore, using a generalized linear model, we aimed to identify alcohol- and smoking-related factors related to telomere length. These statistical analyses were performed using R studio (ver 2022.07.2 + 576), with p < 0.05 considered indicative of statistical significance.

### Ethical approval

This study was approved by the Institute of Tropical Medicine, Nagasaki University (accession number: 201223248-2, date: 20th Jan 2021) and (accession numbers: 210603258, date: 11th Jun 2021). All methods were carried out in accordance with the Declaration of Helsinki.

### Consent for publication

Informed consent was obtained from all the participants who answered the questionnaire and provided blood samples.

## Results

### Telomere length comparison between AUDG and CONT

**Figure 1:**
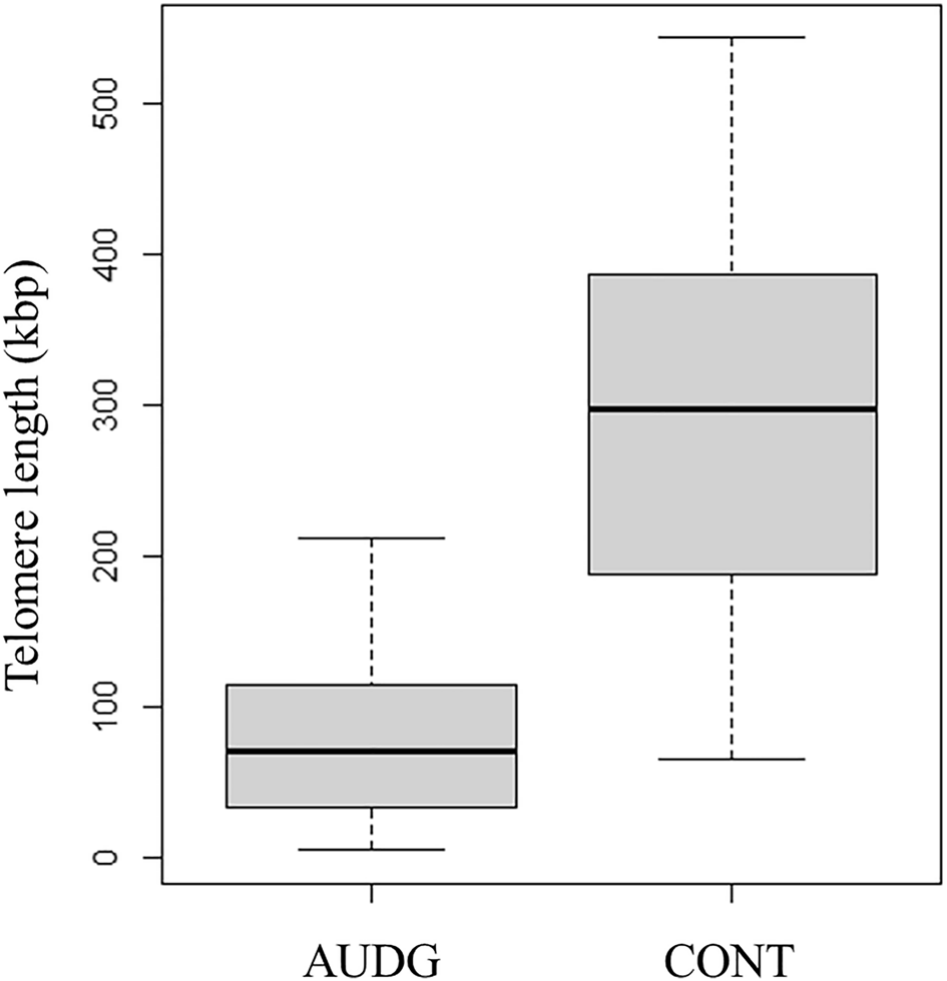
Comparison of telomere length between AUDG and CONT. Telomere length was compared by the Wilcoxon rank sum test, and the AUDG had a significantly shorter telomere length (p < 0.001).

The final numbers of subjects were 74 for AUDG and 68 for CONT. Figure 1 shows the distribution of telomere length in AUDG and CONT. Compared to CONT, AUDG had significantly shorter telomere lengths (p < 0.001). Wilcoxon’s rank sum test confirmed that there was no age difference between the two groups (p = 0.8142).

### Factors correlated with telomere length in AUDG

To search for factors associated with telomere length in AUDG, we first plotted the relationship between each factor and telomere length (Supplementary Fig. S1). This is an auxiliary result, as it only shows the relationship without considering age. As a result of calculating the correlation coefficient of MIC, the value was highest in the smoking duration (r = 0.2744), and telomere length tended to be shorter with more smoking years. On the other hand, a generalized linear model was used to analyze the relationship between each factor and telomere length considering the effect of age (Table 1). Although the number of years of smoking showed a negative correlation with telomere length, the result was not significant (p = 0.055). No other factors were significantly associated with telomere length.

**Table 1:** Relationship between each factor considering age and telomere length.

	Estimate	Std. error	95% CI	*p* value
Intercept	7.34E−03	1.21E−01	[− 2.34E−01, 2.49E−01]	0.952
Age	− 1.25E−01	1.22E−01	[− 3.68E−01, 1.15E−01]	0.308
BMI	4.76E−02	1.22E−01	[− 1.97E−01, 2.92E−01]	0.698
Intercept	− 6.42E−17	1.17E−01	[− 2.33E−01, 2.33E−01]	1.000
Age	− 1.23E−01	1.25E−01	[− 3.72E−01, 1.25E−01]	0.325
Heavy drinking duration	− 1.18E−02	1.25E−01	[− 2.60E−01, 2.37E−01]	0.925
Intercept	− 6.40E−17	1.17E−01	[− 2.33E−01, 2.33E−01]	1.000
Age	− 1.25E−01	1.30E−01	[− 3.84E−01, 1.33E−01]	0.338
Abstinence duration	− 4.80E−03	1.30E−01	[− 2.63E−01, 2.54E−01]	0.971
Intercept	− 5.31E−17	1.15E−01	[− 2.30E−01, 2.30E−01]	1.000
Age	− 6.20E−02	1.25E−01	[− 3.11E−01, 1.87E−01]	0.621
Brinkman index	− 1.78E−01	1.25E−01	[− 4.27E−01, 7.09E−02]	0.158
Intercept	− 5.48E−17	1.14E−01	[− 2.27E−01, 2.27E−01]	1.000
Age	− 1.27E−02	1.29E−01	[− 2.70E−01, 2.44E−01]	0.922
Smoking duration	− 2.52E−01	1.29E−01	[− 5.08E−01, 5.24E−03]	0.055

The results of generalized linear model analysis with age and each factor as explanatory variables and telomere length as objective variables are shown.

**Figure 2:**
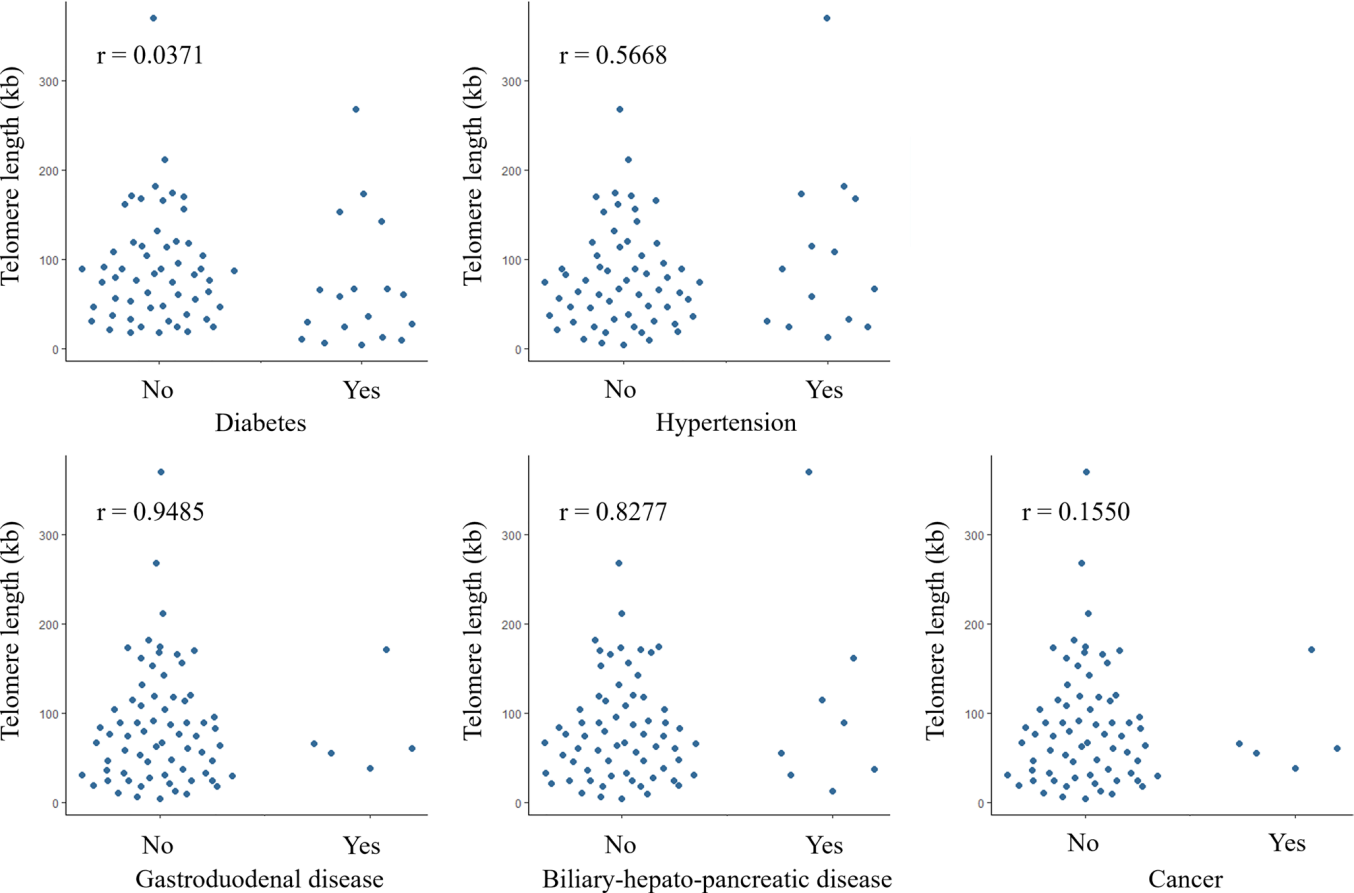
Relationship between morbidity of alcohol-related diseases and telomere length. For each disease, telomere length was compared between the group with a history of disease and the group without a history of disease using the Wilcoxon rank sum test.

Next, as a result of comparing the telomere length between the groups with and without a history of suffering from each disease, telomere length was significantly shorter in the group with diabetes than in the group without diabetes (p = 0.0371) (Fig. 2). On the other hand, there was no significant difference in Heavy Drinking Duration, Brinkman Index, or Smoking Duration depending on the presence or absence of a history of diabetes (Supplementary Fig. S2). No significant association was found between a history of other diseases and telomere length. Considering the effect of age on telomere length, it was confirmed whether there was a difference in age between a group with and without a history of suffering from each disease. As a result, the group with a history of cancer tended to be older than the group without a history of cancer, but the difference was not statistically significant (p = 0.0606) (Supplementary Table S1). In other words, age did not affect the finding that telomere length was significantly shorter in those with a history of diabetes.

Finally, multivariate analysis was performed to evaluate whether physical indices such as age and BMI and indices of exposure duration to drinking and smoking (heavy drinking duration, smoking duration, and abbreviation duration) were associated with telomere length. A longer smoking duration resulted in a significantly shorter telomere length (p = 0.0129) (Table 2).

**Table 2:** Associations of attributes and exposure duration of alcohol and tobacco with telomere length.

Variables	Estimate	Std. error	95% CI	*p* value
Intercept	1.38E−02	1.18E−01	[− 2.22E−01, 2.50E−01]	0.9074
Age	3.90E−02	1.75E−01	[− 3.11E−01, 3.89E−01]	0.8245
BMI	− 6.54E−02	1.32E−01	[− 3.28E−01, 1.97E−01]	0.6206
Heavy drinking duration	5.05E−02	1.65E−01	[− 2.79E−01, 3.80E−01]	0.7602
Smoking duration	− 3.90E−01	1.53E−01	[− 6.95E−01, − 8.56E−02]	0.0129
Abstinence duration	9.60E−04	1.76E−01	[− 3.50E−01, 3.52E−01]	0.9957

A generalized linear model was used to multivariately analyze the effects of age, BMI, duration of alcohol exposure, and duration of tobacco exposure on telomere length.

## Discussion

Similar to previous studies, telomere length was significantly shorter in alcohol use disorder patients than in healthy control subjects in this study^9,16^. Focusing on the factors that shorten telomeres, it has been reported that AUD patients with liver damage and higher γ-glutamyl transpeptidase (γ-GTP) and aspartate aminotransferase (AST) have shorter telomere lengths^17,18^. Thus, the direct effect of alcohol consumption on telomere length has been evaluated. On the other hand, in this study, a long smoking duration and diabetes were also shown to be factors that shortened telomere length among AUD patients. Although many factors, including smoking and diabetes, have each been independently reported as risk factors for telomere shortening^19,20^, we found that telomere shortening in AUD patients may occur by these two factors in the study population. In other words, it was suggested that even smoking habits and comorbid diabetes, which are not directly related to the pathology of AUD, significantly contributed to telomere shortening in AUD patients. Previous studies have reported that telomere shortening is attenuated in diabetic patients under controlled conditions compared to those with uncontrolled diabetes^12^. In this study, we observed a trend toward further telomere shortening in AUD patients with a history of diabetes, suggesting that the impact of having diabetes on telomere length may persist throughout life, even when diabetes is currently under control.

In fact, according to the WHO, smoking contributes more to the global burden of disease than alcohol^21^. The smoking prevalence among AUD patients in the United States (37.84%) is approximately twice that of non-AUD patients (16.29%). They also found that patients with more severe AUD symptoms were more likely to smoke^22^. A study investigating the association between alcohol consumption and the incidence of chronic obstructive pulmonary disease (COPD) reported that moderate drinking was associated with a lower incidence of COPD^23^. Thus, people who drink more may smoke more and have shorter telomeres.

Pancreatic islet β-cells normally contribute to glycemic control by releasing sufficient insulin. However, heavy drinking suppresses insulin secretion and reduces insulin sensitivity, increasing the risk of glucose intolerance^24^. Furthermore, a meta-analysis of nine cohorts (5759 people) reported that diabetes significantly shortened telomere length^25^.

A survey conducted in the Scandinavian countries of Denmark, Finland, and Sweden also reported that the average life expectancy may have been shortened by more than 20 years due to liver damage caused by alcoholism^26^. From our findings, it is particularly important to carry out a smoking cessation program in parallel with the process of treatment and abstinence from alcohol and to control diabetes in cooperation with the internal medicine department to suppress the shortening of healthy life expectancy of AUD patients.

However, it is also necessary to pay attention to differences between countries and ethnic groups. Although this study showed the results of a survey targeting Japanese population, treatment rates for AUD are reported to be low in low- and lower-middle-income countries, but even in developed countries, only 7% of patients in the USA^27^ and 10% of patients in Europe are treated with medication or psychotherapy^28^. Globally, only 17.3% of patients with AUD receive treatment^29^. It has also been reported that only 5% of AUD patients in Japan seek medical advice or treatment^6^. Therefore, there is a need to find patients who are isolated from society without treatment or to create an environment in which it is easy to seek help. There is a need for greater global access to treatment programs for sobriety, regardless of a country’s economic strength. In addition, focusing on ethnic variations in telomere length, it has been reported that individuals of African and Chinese ancestry tend to have longer telomeres compared to white Europeans^30^. It is important to accumulate research findings evaluating the relationship between various AUDs and telomeres while considering these social and biological differences.

Limitations

Several factors influence telomere length, including socioeconomic status and maternal stress, which can impact fetal telomere length^16,31^. Therefore, understanding the relationship between alcohol use disorder (AUD) and telomere length requires considering socioeconomic factors, psychological stress, family history of psychiatric disorders associated with AUD, and cultural aspects. While diabetes in our study participants is medically managed, the current blood glucose levels and their association with telomere length were not assessed. It’s crucial to examine not just diabetes history but also real-time blood glucose trends’ impact on telomeres. Telomere length varies due to genetic and environmental factors, even within specific conditions like AUD. Given our study's limited sample size, further epidemiological research across diverse regions is necessary for comprehensive insights.

Several factors influence telomere length, including socioeconomic status and maternal stress, which can impact fetal telomere length. Therefore, understanding the relationship between alcohol use disorder (AUD) and telomere length requires considering socioeconomic factors, psychological stress, family history of psychiatric disorders associated with AUD, and cultural aspects. While diabetes in our study participants is medically managed, the current blood glucose levels and their association with telomere length were not assessed. It's crucial to examine not just diabetes history but also real-time blood glucose trends’ impact on telomeres. Telomere length varies due to genetic and environmental factors, even within specific conditions like AUD. Given our study's limited sample size, further epidemiological research across diverse regions is necessary for comprehensive insights.

## Conclusions

We investigated the correlation between telomere length and risk factors, including drinking and smoking history as well as medical disease history, among patients with AUD. Our analysis identified specific factors associated with telomere shortening in this target group. While treatment and support for alcohol misuse are commonly available for individuals with AUD, our study highlights the importance of enhancing support for smoking cessation and managing diabetes to enhance patient outcomes. Our research underscores that among individuals grappling with alcohol dependence, prolonged smoking habits and the presence of diabetes are significant contributors to telomere shortening. While interventions and support for alcohol abstinence have traditionally been the focus for patients with alcohol use disorder, our findings suggest the potential efficacy of incorporating smoking cessation programs and diabetes management into comprehensive treatment strategies. This holistic approach may help mitigate the erosion of healthy life expectancy among individuals with alcohol use disorder.

### Supplementary Information

Supplementary Figures.

Supplementary Table S1.

## Data Availability

The datasets used and/or analysed during the current study available from the corresponding author on reasonable request.

## Abbreviations

AUD: Alcohol use disorder
AUDG: Alcohol use disorder patient group
AST: Aspartate aminotransferase
BMI: Body mass index
CONT: Control group
COPD: Chronic obstructive pulmonary disease
γ-GTP: γ-Glutamyl transpeptidase
MIC: Maximum information coefficient
SD: Standard deviation
WHO: World Health Organization
