# Supplementary material for: Smoking and diabetes cause telomere shortening among alcohol use disorder patients
Source: Sci Rep. 2024 Feb 26;14:4701. doi: 10.1038/s41598-024-55195-2 (PMC10897475; doi:10.1038/s41598-024-55195-2)
Supplement: Supplementary file 1 — Supplementary Figures. [file 41598_2024_55195_MOESM1_ESM.docx]

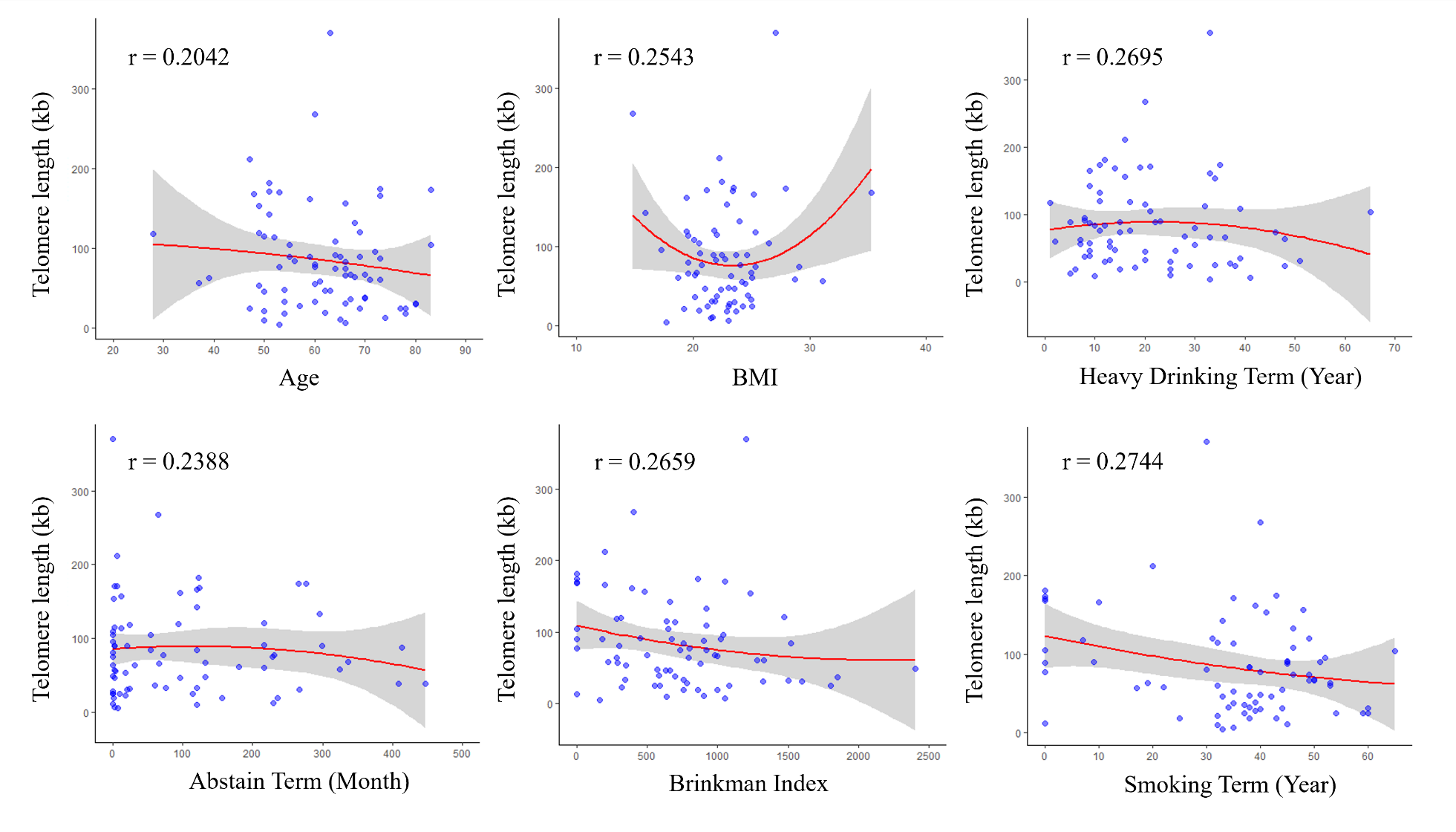
Supplementary figure S1. Relationship between telomere length and attributes, drinking habits, and smoking habits.

The relationship between each factor and telomere length was plotted, and the approximation line and SD of the quadratic equation are shown. Correlation coefficients for MICs were calculated to better reflect the correlation coefficients of the curves.


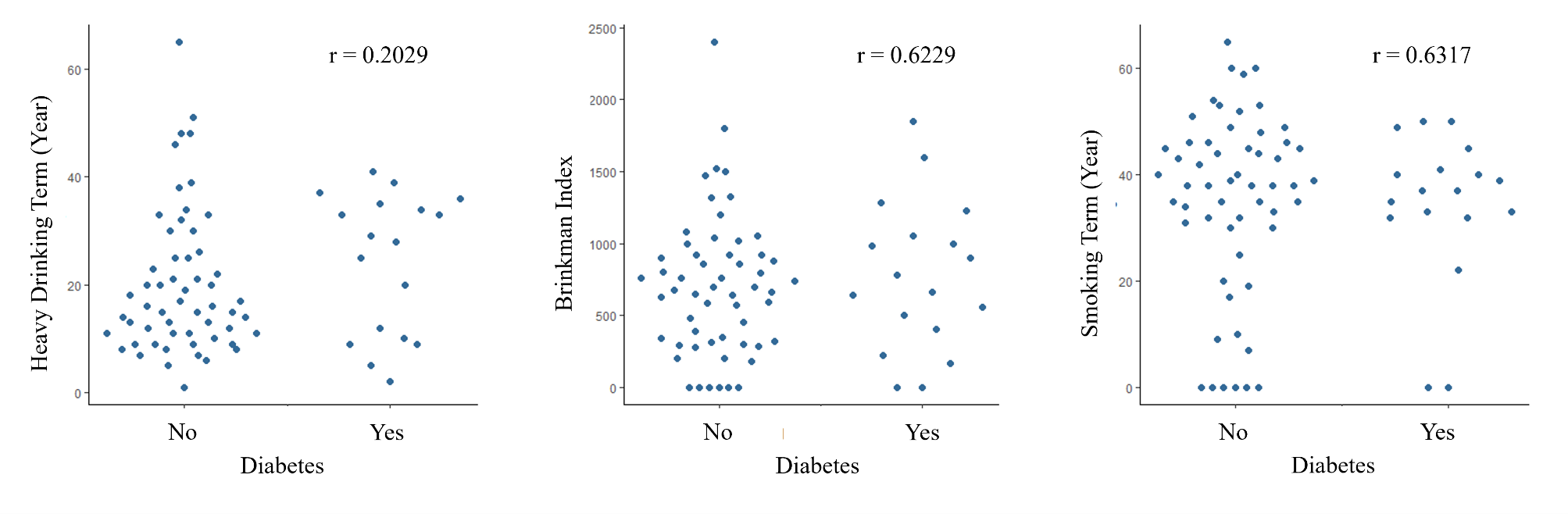


Supplementary figure S2. Association between history of heavy drinking/smoking and prevalence of diabetes.

The values of each factor were plotted and compared by Wilcoxon's rank sum test in the groups with and without a history of diabetes.
